# A novel fed-batch based cultivation method provides high cell-density and improves yield of soluble recombinant proteins in shaken cultures

**DOI:** 10.1186/1475-2859-9-11

**Published:** 2010-02-19

**Authors:** Mirja Krause, Kaisa Ukkonen, Tatu Haataja, Maria Ruottinen, Tuomo Glumoff, Antje Neubauer, Peter Neubauer, Antti Vasala

**Affiliations:** 1 BioSilta Oy, c/o Department of Biochemistry, University of Oulu, Finland; 2 Fachgebiet Bioverfahrenstechnik, Institut für Biotechnologie, Technische Universität Berlin, Ackerstrasse 71-76, Berlin, Germany; 3 Department of Biochemistry, University of Oulu, Oulu, Finland

## Abstract

**Background:**

Cultivations for recombinant protein production in shake flasks should provide high cell densities, high protein productivity per cell and good protein quality. The methods described in laboratory handbooks often fail to reach these goals due to oxygen depletion, lack of pH control and the necessity to use low induction cell densities. In this article we describe the impact of a novel enzymatically controlled fed-batch cultivation technology on recombinant protein production in *Escherichia coli *in simple shaken cultures.

**Results:**

The enzymatic glucose release system together with a well-balanced combination of mineral salts and complex medium additives provided high cell densities, high protein yields and a considerably improved proportion of soluble proteins in harvested cells. The cultivation method consists of three steps: 1) controlled growth by glucose-limited fed-batch to OD_600 _~10, 2) addition of growth boosters together with an inducer providing efficient protein synthesis within a 3 to 6 hours period, and 3) a slow growth period (16 to 21 hours) during which the recombinant protein is slowly synthesized and folded. Cell densities corresponding to 10 to 15 g l^-1 ^cell dry weight could be achieved with the developed technique. In comparison to standard cultures in LB, Terrific Broth and mineral salt medium, we typically achieved over 10-fold higher volumetric yields of soluble recombinant proteins.

**Conclusions:**

We have demonstrated that by applying the novel EnBase^® ^Flo cultivation system in shaken cultures high cell densities can be obtained without impairing the productivity per cell. Especially the yield of soluble (correctly folded) proteins was significantly improved in comparison to commonly used LB, Terrific Broth or mineral salt media. This improvement is thought to result from a well controlled physiological state during the whole process. The higher volumetric yields enable the use of lower culture volumes and can thus significantly reduce the amount of time and effort needed for downstream processing or process optimization. We claim that the new cultivation system is widely applicable and, as it is very simple to apply, could widely replace standard shake flask approaches.

## Background

The widely used standard system for shake flask cultures is the "Sambrook protocol" described in *Molecular cloning *laboratory manual [1]. This protocol has already been used over two decades, often quite successfully, to produce recombinant proteins in *E. coli*. Proteins that fail to be produced with this protocol are regarded as difficult and to require special tricks for production. The Sambrook protocol has however several limitations. Successful protein production requires induction of recombinant protein synthesis in the middle of exponential growth when the growth rate is highest. This inevitably means that fairly low induction cell densities are used, and consequently, due to the impact of recombinant protein production on the cellular maintenance, the resulting cell densities remain quite low. With this method large culture volumes are needed to produce enough material for protein structural studies for example. An ideal system for recombinant protein production would allow for both high cell densities and high protein productivity per cell, but development of such a cultivation system for shaken cultivations is not a trivial task.

### What happens in shake flasks?

Shake flask cultures are normally run in batch mode, i.e. all culture components are already added at the start of the cultivation, without monitoring and control of any parameters such as pH or the level of dissolved oxygen. Under such circumstances, high cell densities cannot be reached since the high respiratory rate of fast growing bacteria exceeds the oxygen transfer capacity of the cultivation vessel, and the culture will relatively soon be depleted of oxygen [2]. Under oxygen limitation growth is slow and recombinant protein production is poor. Oxygen limitation leads to induction of over 200 genes connected to anaerobic responses [3,4], which multiply the stress caused by the recombinant protein synthesis. Moreover, the appearance of anaerobic conditions easily leads to spoilage of the culture medium by anaerobic metabolites. In addition to oxygen limitation, uncontrolled batch cultures may suffer from acetate accumulation by overflow metabolism. The drop of pH due to accumulation of overflow acetate and anaerobic metabolites impairs cell growth and leads to poor recombinant product formation. Moreover, pH maintenance of shaken cultures is further hampered by metabolic events such as utilization of peptides as a carbon source (pH increases since the excess ammonia is secreted into the medium) and consumption of ammonia-containing chemicals in mineral salt medium (decreases pH). It is worthy of note that acetate and other acidic metabolites exhibit growth-inhibitory effects regardless of medium pH [5,6], and therefore the detrimental effects of overflow metabolism and oxygen limitation cannot be completely addressed by adequate medium buffering.

### Conventional approaches to improve protein production

Uncontrolled growth is often associated with uncontrolled protein synthesis rate, incorrect protein folding and metabolic fluctuations due to variations in the intracellular levels of free amino acids. Very strong promoters (lambda P_L _and P_R_, P_tac_, P_trp_, P_T7_) are almost exclusively used in recombinant protein production. Therefore protein production cannot be efficiently controlled at the level of mRNA synthesis. Also the control of protein translation is challenging, since the number of ribosomes in the cell varies according to the physiological state so that their number is highest during the active growth. High local concentration of recombinant protein together with insufficient amount of folding-promoting proteins may lead to formation of insoluble protein aggregates (inclusion bodies). This problem is commonly addressed by reducing the protein synthesis rate by the use of lower IPTG concentrations [7] and/or lower induction temperatures.

### Medium optimization

Increase in cell density can be achieved in shaken cultures by the choice and optimization of cultivation medium. In short-term shaken cultures, different combinations of yeast extract and peptones are favored due to easy preparation and availability of essential growth factors and vitamins. Luria-Bertani (LB) medium is a typical example of such media. Higher cell densities can be reached with media that have even higher nutrient contents and improved buffering, like Terrific Broth [8]. Mineral salt media, although preferred for bioreactor cultivations, are not widely used for recombinant protein production in shaken cultures due to lower cell yield compared to rich cultivation media. The drawback of mineral salt media is that some of the medium components cannot be supplied in sufficient concentrations to support growth to high cell densities, since such high concentrations would be toxic to the cells (e.g. ammonia) or result in precipitation (e.g. magnesium). In bioreactor cultivations such components can be easily provided by constant or intermittent feeding, but in shaken cultures this is often practically impossible.

### Lessons to learn from bioreactors

In bioreactors, *E. coli *can be successfully cultivated to high cell densities by applying the fed-batch principle, which has been widely utilized since late 1980's [9-11]. A substrate-limited fed-batch is a cultivation technique which comprises a batch process continuously fed with a substrate solution so that one substrate component is growth rate limiting. In this way, the growth rate can be controlled to match the oxygen transfer rate, allowing for the cultivation to be run in aerobic cultivation mode. As the substrate feed solution must be very concentrated to reach a high cell density, application of the fed-batch technique in shaken cultures has until lately been practically impossible. In the recent years some novel technologies to provide feeding without external devices have been developed. For example, Jeude et al. [12] have developed a system where glucose is gradually released from silicone polyelastomer discs, and a second system developed in the University of Aachen [13] is based on slow leakage of a concentrated nutrient solution through a polymer network providing a kind of "dialysis bioreactor" condition. In a glucose release system developed in the University of Oulu, glucose is enzymatically released to the culture from starch that has been immobilized into a starch/agar gel [14]. This enzymatic glucose release principle has now been developed into a fully liquid phase cultivation system, EnBase Flo, where the glucose-releasing polymer is fully soluble and no gel phase is necessary (see http://www.biosilta.com for details).

In this article we demonstrate, by presenting three different cases of recombinant protein production in different cultivation formats (shake flasks and 24-deep well plates), that the novel EnBase Flo system provides high cell densities without a decrease in recombinant protein productivity per cell. The A-domain of human protein disulphide isomerase (PDI) is a 15 kDa protein performing disulphide bond isomerisation in the human ER. Alcohol dehydrogenase (ADH) from *Lactobacillus *(approximately 31 kDa) has been earlier shown to be well produced both in bioreactor cultivations and in shake flasks [15] in an expression system based on pQE30-plasmid. It is a R-specific enzyme requiring NADP(H) as a coenzyme and has been successfully used for industrial-scale whole-cell biotransformations for preparation of stereoselective chemicals. Multifunctional enzyme type 2 from *Drosophila melanogaster *(Dm_MFE-2) is a multidomain protein exhibiting two enzymatic activities: an NADH-dependent (3*R*)-hydroxyacyl-CoA dehydrogenase and a 2-enoyl-CoA hydratase 2 implicated in fatty acyl β-oxidation. The size of the native protein is 64 kDa, and together with an affinity tag fusion the total size is 72 kDa. Although it does not contain many disulphide bonds, the large size makes it difficult to produce in soluble form. In this study we express these three proteins in EnBase Flo, conventional LB, TB and mineral salt medium. By examination of cell growth, medium pH development and protein yields we demonstrate that significant improvements in protein yield can be achieved by this new cultivation method.

## Results

### Cultivation characteristics

**Figure 1:**
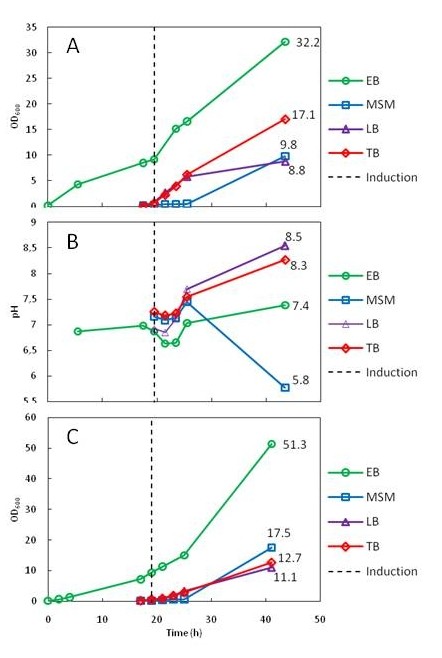
**Characteristics of recombinant *E. coli *strain RB791 [pQE30:Adh] cultivations in different culture media**. EB = EnBase Flo medium, MSM = mineral salt medium, LB = Luria-Bertani medium, TB = Terrific Broth medium. A: OD_600 _in 50 ml cultures in 500 ml shake flasks; B) pH in 50 ml cultures in 500 ml shake flasks; C) OD_600 _in 3 ml cultures in 24-deep well plates. The time point of induction (0.4 mM IPTG) is indicated with a dashed vertical line. EnBase Flo cultures were also supplemented with "medium booster" (complex additives) at the same time with induction. EnBase Flo cultures were grown for 19 h before induction, whereas cultures in MSM, LB and TB media were grown for 2 h before induction. All cultures were run until 24 h after induction.

The results from 3 ml cultures in 24-deep well plates (24DWPs) and 50 ml cultures in 500 ml shake flasks demonstrate that continuous growth to high cell densities and maintenance of favorable pH can be achieved with EnBase Flo (Figure 1, Table 1). The growth characteristics and medium pH conditions during recombinant protein production by *E. coli *strains RB791 [pQE30:adh], BL21(DE3) [pPal7:Dm_MFE-2] and BL21(DE3) pLysS [pET23:pdi] in different cultivation media are summarized in Table 1. Figure 1 presents the detailed OD_600 _and pH data for one of the strains (RB791 [pQE30:adh]). The different media had remarkably different pH maintenance capacities. While EnBase Flo was capable of maintaining pH between 6.5 and 7.5 throughout the 43 h cultivation, pH in mineral salt medium (with 10 g l^-1 ^glucose) tends to drop (especially in the shake flask cultivation, where final pH was below 6), and in LB and TB media pH rose above 8 during prolonged cultivation (Figure 1, Table 1). EnBase Flo was superior to the other media also with respect to cell density. Depending on the strain, final OD_600 _(at 43 h) in EnBase Flo cultures was 32 to 51 in 24DWP and 25 to 32 in shake flasks, whereas in mineral salt medium, LB and TB cultures final OD_600 _(at 26 h) were 2 to 17, 4 to 11 and 10 to 17, respectively (Figure 1, Table 1).

**Table 1:** Growth and pH characteristics in all cultivations. after induction) are presented.

Strain	Cultivation format	Medium	OD_600 _at ind.	pH at ind.	OD _600_at harvest	pH at harvest
RB791 [pQE30:adh]		LB	0.4	7.1	11.1	8.6
	24DWP	TB	0.6	7.0	12.7	8.4
		MSM	0.2	7.2	17.5	6.8
		Enbase Flo	9.3	7.0	51.3	6.9

BL21 (DE3) [pPal7:Dm_MFE-2]		LB	0.6	7.2	5.9	8.0
		TB	0.8	6.9	10.1	8.4
	24DWP	MSM	0.2	6.9	12.6	6.9
		Enbase Flo	12.1	6.9	31.7	7.2

BL21(DE3) pLysS [pET23:pdi]		LB	0.2	6.8	6.1	8.6
		TB	0.5	7.0	16.9	8.5
	24DWP	MSM	0.2	7.2	2.5	6.8
		Enbase Flo	12.1	7.0	33.3	7.3

**Strain**	**Cultivation format**	**Medium**	**OD_600 _at ind.**	**pH at ind.**	**OD _600_at harvest**	**pH at harvest**

RB791 [pQE30:adh]		LB	0.9	6.9	8.8	8.5
	shake flask	TB	0.7	7.3	17.1	8.3
		MSM	0.3	7.2	9.8	5.8
		Enbase Flo	9.2	6.9	32.2	7.4

BL21(DE3) [pPal7:Dm_MFE-2]		LB	0.7	6.9	4.4	8.5
	shake flask	Enbase Flo	12.8	6.8	24.7	6.6

OD_600 _and pH at induction and at final cell harvest (24 h

### Recombinant protein production

**Figure 2:**
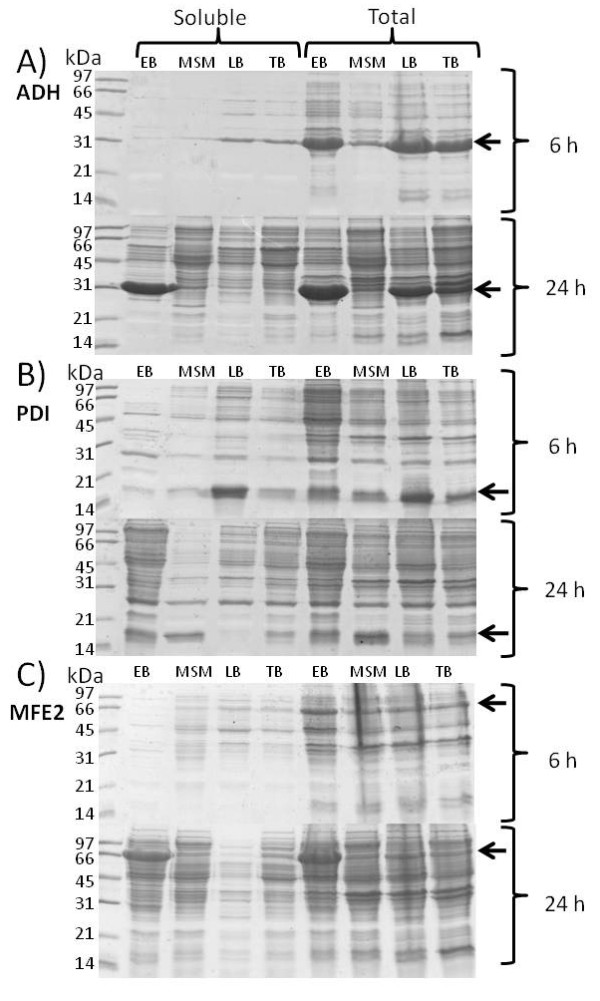
**Recombinant protein production in 24 DWPs using different cultivation media**. A) Stereoselective R-alcohol dehydrogenase (ADH) from *Lactobacillus *produced by *E. coli *RB791 [pQE30:Adh]; B) Protein disulphide isomerase A-domain (PDI) produced by *E. coli *BL21(DE3)pLysS [pET23:pdi]; C) Multifunctional enzyme type 2 of *Drosophila *produced by *E. coli *BL21(DE3) [pPal7:Dm_MFE-2]. In each sub-picture (A, B, C) soluble proteins are shown on the left and total proteins on the right. The upper and lower parts of the pictures present proteins after 6 h and 24 h induction, respectively. To facilitate direct comparison between different cultivation media, all samples were diluted to equal cell concentration before cell lysis and loading on gel. EB = EnBase Flo medium, MSM = Mineral Salt Medium, LB = Luria Bertani, TB = Terrific Broth. Target protein is indicated by arrows.

**Figure 3:**
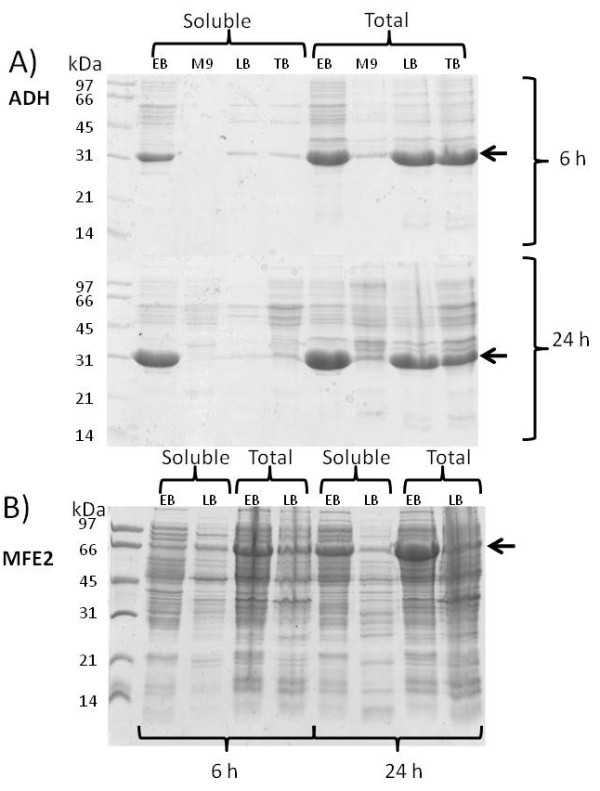
**Recombinant protein production in 50 ml cultures in 500 ml shake flasks using different cultivation media**. A) Stereoselective R-alcohol dehydrogenase (ADH) from *Lactobacillus *produced by *E. coli *RB791 [pQE30:Adh]; B) Multifunctional enzyme type 2 of *Drosophila *produced by *E. coli *BL21(DE3) [pPal7:Dm_MFE-2]. Each sub-picture presents soluble and total proteins after 6 h and 24 h induction. To facilitate direct comparison between different cultivation media, all samples were diluted to equal cell concentration before cell lysis and loading on gel. EB = EnBase Flo medium, MSM = Mineral Salt Medium, LB = Luria Bertani, TB = Terrific Broth. Target protein is indicated by arrows.

**Figure 4:**
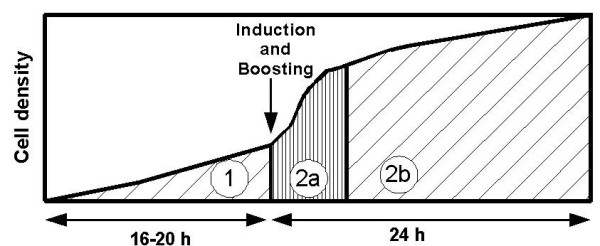
**A schematic picture of the EnBase^® ^Flo cultivation principle**. Phase 1 = controlled cultivation in mineral salt medium with a low concentration of complex additives (peptones, yeast extract). Phase 2 = induction with IPTG and addition of complex nutrients (booster mixture). Phase 2a of increased growth rate is followed by Phase 2b of low growth rate and slow production of soluble protein.

SDS-PAGE gels with total and soluble protein at 6 h and 24 h after induction are presented in Figures 2 and 3. When a 24 h induction period was applied, the productivity per cell of total and especially soluble protein was remarkably higher in EnBase Flo than in other media in *E. coli *strains RB791 [pQE30:adh] and BL21(DE3) [pPal7:Dm_MFE-2]. Combined with the higher cell density in EnBase Flo, these results indicate many-fold increased volumetric recombinant protein productivity for EnBase Flo in comparison to the other media. Since the proportion of soluble protein in EnBase Flo cultures increased substantially from 6 to 24 hours (Figures 2 and 3), it seems probable that during the slow growth (phase 2b in Figure 4) insoluble recombinant proteins are partly replaced with soluble proteins. With mineral salt medium, LB or TB, prolonged induction time did not increase the yield per cell or the proportion of soluble protein. With the *E. coli *strain BL21(DE3)pLysS [pET23:pdi], highest total and soluble productivity per cell was achieved in LB medium with 6 h induction time. It should be noted, however, that there OD_600 _after 6 h induction was only 0.8 (data not shown), whereas OD_600 _in EnBase Flo cultures after 24 h induction was 33. Therefore, the volumetric productivity of EnBase Flo is likely to outperform LB also in this case due to 40-fold higher cell density. Extending the induction period up to 24 h in LB medium resulted in increased cell density up to OD_600 _of 6, but the productivity per cell decreased remarkably, suggesting extensive protein degradation or upgrowth of plasmid-free cells.

The impact of the new cultivation method on the yield of purified soluble protein was evaluated with the MFE-2 protein (72 kDa) produced in a shake flask (purification data not shown). The previously used cultivation system (6× 1000 ml cultures in LB medium) had yielded to 8 mg of crystallization grade protein (1.3 mg per 1 liter medium), whereas a 400 ml EnBase Flo cultivation yielded 6.7 mg of pure protein (17 mg per 1 liter medium). Hence EnBase Flo gave over 13-fold higher amount of crystallization grade protein per volume even without taking into account that the capacity of the Profinity eXaxt columns used for EnBase Flo culture material is much lower than that of the previously used Ni-NTA columns applied for purification of LB culture material.

## Discussion

The novel cultivation system described here combines an enzymatic glucose release system with an optimized medium (mixture of mineral salts and complex additives) in a way which provides high cell densities together with high recombinant protein yields in shaken cultures. Cell growth can be controlled and a favorable pH level can be maintained during the whole cultivation. Due to the growth control, accumulation of harmful metabolites can be minimized and appearance of anaerobic conditions can be avoided. The cultivation takes one day longer to perform compared to the standard cultivation system, but regarding the "hands-on time" it is even easier and faster to perform. Since the cultivation volumes can be substantially decreased, several cultivations (e.g. many strains or an optimization set) can be performed at the same time. The possibility to use lower cultivation volumes also enables the use of microwell plates or deep well plates, which brings new applications for robot-driven automated cultivations.

With EnBase Flo, 10 to 20-fold higher induction cell density could be used, and the final cell densities were typically 10 times higher compared to the standard Sambrook protocol. This increase was coupled with equal or improved recombinant protein productivity per cell. The higher yield of soluble protein fraction is probably due to the extended protein synthesis period (24 h) under favorable conditions. These described improvements were achieved at a cultivation temperature of 30°C. There was no need to use lower temperatures, since optimal conditions for a long induction and controlled growth were obtained by the new cultivation technology. Cultivation temperatures as high as 37°C have also been applied with EnBase Flo and found to provide high soluble protein yield (data not shown). However, we have chosen to generally use 30°C for EnBase Flo cultures due to higher oxygen solubility and less evaporation. Also the number of functional ribosomes in the cell is known to be higher at lower temperatures [16]. It is also possible that the overall changes in cell physiology occurring at fed-batch cultivation mode and elevated cell density may promote improved protein folding, possibly through increased synthesis of folding-assisting proteins. Protein yield of EnBase Flo cultures was relatively high already after 6 h induction, but often most of it was in insoluble form. This suggests that the bacterial growth rate immediately after boosting (i.e. addition of complex nutrients) was not low enough to provide correct protein folding. However, when a longer induction time (24 h) was applied, the protein produced was usually in soluble form, which was not the case with prolonged induction in MSM, LB and TB media.

An increased proportion of soluble protein by the use of the enzyme-based fed-batch technology has been earlier demonstrated by Panula-Perälä et al. [14]. They reported lower total productivity per cell in their system compared to standard medium (M9 in that case), but achieved 8-10 times increased volumetric protein yield with EnBase due to the drastically higher cell density, and a higher proportion of TIM (triosephosphate isomerase) in a soluble form. Although the slow growth during pre-induction phase may pre-adapt the cell to stress [17], it also adjusts the protein synthesis system to a very low activity. This may explain why the advantage of high cell yield could not be directly translated to high recombinant protein productivity in the system described by Panula-Perälä et al. Therefore some major improvements were made to reach the good performance of the system described in this article. The new system is not anymore based on plain mineral salt medium composition. The utilization of a low amount of complex medium additives for the first overnight cultivation results in a slightly higher cell yield together with an improved pH balance. Also the addition of a high dose of complex nutrients ("boosting") at the time of induction provides a sufficient supply of the key medium compounds (e.g. amino acids, trace metals, cofactors and vitamins) needed for the setup of efficient protein synthesis. Beneficial effects of complex additives have been earlier reported by some bioreactor studies. For example, Tsai et al. [18] reported a 10-fold increase in intracellular human IGF-1 accumulation by the addition of yeast extract and tryptone. There are also some indications that product stability may be improved by the use of complex additives. Swartz [19] hypothesized in a review on recombinant DNA technology that complex additives may also reduce product proteolysis. It is however possible that instead of actually reducing proteolysis, complex nutrients might just compensate for it due to prolonged maintenance of efficient protein synthesis. This view is supported by our observation that an extended protein synthesis period results in higher amount of soluble recombinant protein.

Detailed information about the cellular mechanisms of *E. coli *during cultivation in EnBase Flo medium is not yet available, but the cultivation supposedly proceeds as follows: bacteria are first cultivated overnight in a balanced medium, where the growth can be tightly controlled by enzymatic glucose feeding (phase 1 in Figure 4). Thereafter, complex nutrients are added ("boosting") at the same time with an inducer to prepare the cells for efficient recombinant protein production. This medium boosting is beneficial since the recombinant protein synthesis has a major effect on growth and cell maintenance [20]. When growth rate is very low most of the metabolic energy is allocated to cell maintenance instead of the recombinant protein synthesis. Additionally, the "boosting" also helps to maintain the required pH. An increase in growth rate is temporarily achieved by the boosting step, and there is approximately 2-fold increase in cell density within 6 hours (phase 2a in Figure 4). Such a moderate increase in biomass implies that despite the presence of rich medium components the growth is not fully uncontrolled. Possibly the enzymatic glucose delivery system keeps the sugar phosphotransferase transport system at least partly saturated [21], thereby decreasing the utilization of alternative carbon sources and allowing some level of growth control. The increase in growth rate after medium boosting is associated with an increase in protein synthesis, which results in efficient recombinant product formation. However, the product formation rate is probably lower than that of the recombinant protein production phase typical for normal shake flask processes in rich media. With prolonged induction (further 18 h, phase 2b in Figure 4) the cell number approximately only doubles, and protein production switches to a transient phase where the total amount of recombinant protein does not very much change in the cell. However, it is probable that new proteins are slowly synthesized while the old, partly insoluble, proteins are gradually degraded or even re-folded. Carrió and Villaverde [22,23] have deduced that only a restricted amount of recombinant proteins can be coprocessed (correctly folded) together with the host proteins. Therefore the availability of folding-promoting proteins (chaperones) may limit the correct folding. In this respect, the lower protein synthesis rate in fed-batch cultivation may be very beneficial. Prior to induction, the slow growth rate near to starvation limit may considerably increase the amount of chaperone-type proteins, and the few cell divisions that occur later possibly do not dilute them away. Also the cell physiology during fed-batch high cell density growth can be very different from the batch growth in shake flask cultures, and especially the higher amount of stress proteins related to low growth rate may affect protein folding.

The fastest growth occurs immediately after the addition of booster and inducer, when the cell number is often doubled within 4 to 6 hours. This growth rate (μ = 0.15 to 0.25) is just slightly higher than the growth rate known to induce starvation response [20], but has been earlier shown to be the optimal growth rate for recombinant protein production. Additionally the complex medium additives in the booster solution provide easily available raw material for protein synthesis. Such a growth rate is much lower than growth rates in the Sambrook process (cell number typically doubled within 20 to 40 minutes). Our results suggest that this reduction in growth rate effectively enhances correct folding of recombinant proteins. The traditional approaches to reduce protein synthesis rate and promote correct folding include the use of low temperatures [24,25], use of low inducer concentrations [7], and the use of extreme stress conditions like low or high pH [26]. Our cultivation method can, however, provide the control of protein synthesis rate at the usual cultivation temperatures (30 or 37°C) and with commonly used IPTG concentrations (0.4 or 1 mM). The strategy to reduce the metabolic load of cells during recombinant protein synthesis can be further applied to EnBase Flo as well as to any other cultivation systems.

The common approaches for pH maintenance during cultivation are based on the use of well-buffered culture media. Although such buffering prolongs the time of efficient bacterial growth, the buffering capacity can be suddenly lost when the amount of H+ or OH- ions exceeds the capacity of the buffer. Furthermore, high concentrations of buffering agents such as 3-(N-morpholino)-propanesulfonic acid (MOPS) and tris-(hydroxymethyl)aminomethane (Tris) may inhibit the growth of bacteria. It should also be noted that no pH buffering system can prevent the accumulation of harmful metabolites such as acetate into the cultivation medium. The pH maintenance system of EnBase Flo is based on another operation principle than buffering system. We have observed that there are remarkable differences between mineral salt media and rich media with respect to the development of pH level (see Table 1 as an example). In mineral salt based media pH tends to drop. This pH drop is not only caused by accumulation of acidic by-products, since it occurs also in fed-batch cultivations, as well as in *E. coli *strains that do not produce substantial amounts of acetic acid, but is due to the consumption of ammonia during the growth. In contrast, in *E. coli *cultivations performed in rich cultivation media the pH usually tends to increase. This is very likely a consequence of the utilization of complex compounds (e.g., yeast extract, protein peptones, casamino acids) as a carbon source. During limitation or absence of glucose or other easily-assimilable carbon sources, *E. coli *uses complex compounds for energy generation, and the useless nitrogen will be secreted into the medium as ammonium ions. Therefore, the use of glucose-limited fed-batch together with an adequate concentration of complex additives provides new possibilities for pH control. The rate of glucose delivery can be optimized together with the amount of complex medium additives to provide a suitable pH level throughout the cultivation in a simple phosphate buffer system.

The shake flask cultivation results described in this article were obtained in normal round-bottom Erlenmeyer flasks. In contrast the Structural Genomics Consortium recommends the use of baffled shake flasks [27] for efficient aeration and cell growth. We, however, recommend the use of ordinary round-bottom Erlenmeyer flasks for the following practical reasons: 1) the EnBase Flo system brings such an efficient growth control that the improvement in oxygen transfer by baffles is not necessary, and 2) improved mixing by baffles is associated with upwards spilling and increased foaming. For a long-term cultivation it is extremely important that the oxygen-permeable membrane or other closure used is always dry, since the wetting of the closure, as well as extensive foaming, can significantly reduce oxygen transfer [28], and therefore the use of baffled flasks can actually be counterproductive with respect to oxygen transfer. Closure type [29] and flask filling volume [30] are factors that have a profound effect on oxygen transfer and consequently the performance of cultures, but are however often not appropriately considered by researchers.

The EnBase Flo cultivation method is not bound to a certain promoter system; it has been successfully used with arabinose- or tetracycline-inducible expression systems (data not shown). Therefore, additional control of protein synthesis can be obtained by the use of weaker promoters (i.e. other than the T7-promoter system). The possibility to exploit extended induction times could demonstrate the benefits of weak promoters much better than the use of standard shake flask cultures. In this way, it may be possible to get relevant data for the development of bioreactor cultivations already in a small scale. The results with *E. coli *RB791 [pQE30:Adh] suggest that the standard shake flask-based recombinant protein production process may favor the T7 expression system. This clone is known to produce high amounts of active ADH in glucose-limited fed-batch in mineral salt medium [15]. The gene *adh *has been cloned under the control of phage c6 promoter which is recognized by the native *E. coli *RNA-polymerase. High yields of soluble ADH were obtained in EnBase Flo cultures, while the other cultivation media exhibited poor production of soluble ADH. Therefore it seems probable that if the expression system for bioreactor cultivations was selected on the basis of shake flask cultivations with ordinary media, such potent expression strains like the strain RB791 [pQE30:Adh] would possibly be discarded and a T7-polymerase based system selected instead.

The scale-up of EnBase Flo cultivations from 3 ml culture volume (in 24 DWP) to 50 ml culture volume (in 500 ml shake flask) didn't cause deterioration of the protein production. This indicates a good scalability of the system. If required, further fine-tuning can be easily achieved by enzyme dosing. The possibility to obtain high cell densities makes EnBase Flo very appealing for screening and automatic sample treatment purposes. It has also provided very good results with leaky expression systems, provided that appropriate precautions are taken in the preparation of the inoculant cultures (data not shown). A cultivation system that provides a high yield of recombinant proteins in soluble form is also invaluable for researchers performing affinity purification of His-tagged proteins, since this scheme relies entirely on the presence of soluble proteins. Further, EnBase Flo could be a powerful tool for growth rate optimization in small scale. Bioreactor process optimization studies have demonstrated that the highest recombinant protein production level is obtained with a certain growth rate[20]. With enzymatic fed-batch technology, this optimal growth rate can be experimentally determined in shake flasks or deep well plates.

## Conclusions

We have demonstrated that by applying the EnBase Flo cultivation system in shaken cultures high cell densities (OD_600 _of 30 to 50) can be obtained, high productivity per cell can be maintained or even improved, and long induction periods bringing significant benefits compared to the use of other cultivation media (LB, TB or MSM medium) can be used. Adequate pH control can be obtained with a balanced medium comprising a simple phosphate buffer. In contrast, the commonly used protocols for shake flask cultivations are restricted by very short induction period, uncontrolled growth and fast protein synthesis which often lead to incorrect protein folding and low yield of biologically active soluble protein. This can be regarded as a significant benefit for researchers who make protein structural analysis or apply affinity purification for their proteins. EnBase Flo cultures can be easily scaled up. The potential for high cell densities and growth control makes the system ideal for high throughput screening purposes in small scale. In conclusion, we claim that everyday laboratory work could be greatly enhanced by replacement of the commonly used shake flask cultivation procedures with this novel cultivation system.

## Methods

### Strains and plasmids

*E. coli *BL21(DE3)pLysS [pET23:pdi] strain expressing the A-domain of human protein disulphide isomerase was obtained from prof. L. Ruddock. The *E. coli *BL21(DE3) [pPal7:Dm_MFE-2] strain expressing multifunctional enzyme type 2 of *Drosophila melanogaster *was constructed by T. Haataja. *E. coli *strain RB791 [pQE30:adh] [15] was obtained from IEP GmbH (Wiesbaden, Germany).

### Cultivation media

Four different media were used for the cultivations: 1) Luria-Bertani (LB) medium; 2) Terrific Broth (TB); 3) mineral salt medium (MSM) with 10 g l^-1 ^glucose; and 4) EnBase Flo medium. Luria-Bertani medium was prepared using LB broth from Sigma, 20 g l^-1^. Terrific Broth medium contained (per liter): 12.0 g tryptone, 24.0 g yeast extract, 4 ml glycerol, 100 ml 0.17 M KH_2_PO_4_, and 100 ml 0.72 M K_2_HPO_4_. The glucose-free mineral salt medium (MSM) had the following composition (per liter): 2.0 g Na_2_SO_4_, 6.12 g (NH_4_)_2_SO_4_, 0.50 g NH_4_Cl, 14.60 g K_2_HPO_4_, 3.60 g NaH_2_PO_4_·H_2_O, 1.00 g (NH_4_)_2_-H-citrate, 3 mM MgSO_4_, 0.1 g thiamine hydrochloride, and 2 ml trace element solution (containing per liter: 0.50 g CaCl_2_·2 H_2_O, 0.18 g ZnSO_4_·7 H_2_O, 0.10 g MnSO_4_·H_2_O, 20.1 g Na_2_-EDTA, 16.70 g FeCl_3_·6 H_2_O, 0.16 g CuSO_4_·5 H_2_O and 0.18 g CoCl_2_·6 H_2_O). For cultivations the MSM medium was supplemented with 10 g l^-1 ^glucose. Cultivations with the novel substrate delivery system, EnBase Flo, were performed based on the aforementioned mineral salt medium, which was further supplemented with complex medium additives (optimized combination of peptones and yeast extracts) and some additional trace elements. Also the concentrations of ammonia-containing chemicals were further optimized. The EnBase Flo system is based on enzymatic release of glucose by glucoamylase enzyme. Additionally, all media contained 0.1 ml l^-1 ^Antifoam 204 (Sigma Aldrich) to prevent foaming and 100 μg l^-1 ^ampicillin to maintain plasmid stability.

### Cultivation conditions

All cultures were inoculated with cells washed with 0.9% NaCl solution from agar plates incubated overnight at 30°C, or with cells from premade glycerol stocks. For the preparation of the glycerol stocks, cells were streaked on agar plates, incubated at 30°C overnight and washed off with mineral salt medium containing 15-20% glycerol. The OD_600 _of the stocks was between 10 and 50. The glycerol stocks were stored at -70°C. All cultures (shake flasks and 24DWPs) were started at an OD_600 _of 0.15.

Shake flask cultures were performed in 500 ml Erlenmeyer flasks containing 50 ml of the appropriate medium at 30°C on an orbital shaker (250 rpm). After inoculation, EnZ I'm (glucoamylase provided with BioSilta products) was added to the EnBase Flo cultivations to concentration 0.6 U l^-1^, and the culture was immediately started. The shake flasks were sealed with breathable membranes (Air-O-Top™, Thomson). Cultivations in 24-deep well plates (Riplate^®^, Ritter GmbH) were incubated at 30°C on an orbital shaker (200 rpm), 3 ml per well. After inoculation, EnZ I'm was added to the EnBase Flo cultivations to concentration 1.5 U l^-1^, and the culture was immediately started. The plates were sealed with breathable membranes (Starlab), and the humidity of the shaker was kept at approximately 80% to minimize evaporation.

After overnight cultivation (18 h, OD_600 _= 9-13), the EnBase Flo cultures were supplemented with a "booster solution", providing additional complex medium additives and 1.5 U l^-1 ^(for shake flasks) or 3 U l^-1 ^(for 24DWPs) glucoamylase, and product synthesis was simultaneously induced by addition of 0.4 mM IPTG. The LB, TB and MSM cultures were induced by 0.4 mM IPTG at 2 hours after starting the cultures (OD_600 _= 0.2-0.9). All cultures were run for 24 hours after the addition of IPTG. Cell growth was followed by optical density (OD) measurements. With samples from shake flask cultures, OD was measured with a spectrophotometer (UV-1601, UV-Visible Spectrophotometer, Fennolab) at 600 nm (OD_600_) in cuvettes, and with samples from deep well plate cultures, OD was measured with a microplate scanning spectrophotometer (PowerWave_X_, Bio-Tek Instruments, US) at 490 nm (OD_490_). One unit of OD_490 _corresponds to 2.51 OD_600 _units. All cell density values are presented as OD_600 _values. One unit of OD_600 _corresponds to a dry cell weight of 0.3 g l^-1 ^[31]. pH of each culture was followed offline with a pH electrode (IQ240, IQ Scientific Instruments).

### Protein Analysis

Samples for protein analysis were harvested at 6 h and 24 h after IPTG addition from all cultures. The samples were pelleted by centrifugation (14000 rpm for 4 min), supernatants were discarded and the pellets were frozen at -20°C. For analysis, the cell pellet was resuspended in Tris buffer (pH 7.5) and disrupted by sonication on ice with a sonotrode of 2 mm diameter and 100% power input (Dr. Hielscher GmbH) in 4 × 30 s periods with 10 s cooling breaks in between. Complete cell disruption was ensured by four freezing (-20°C) and thawing (RT) cycles. To obtain the soluble protein fraction, the disrupted cell solution was centrifuged at 14000 × g (4 min, +4°C), and an aliquot for soluble protein analysis was drawn from the supernatant. Total and soluble protein samples were analyzed by SDS-PAGE. The bands were visualized by Coomassie Brilliant Blue staining. Prior to cell lysis the volume of Tris buffer per each sample was adjusted so that the resulting cell density was the same in all samples. Therefore, protein productivity per cell in different samples can be directly compared on the basis of the protein band thickness.

## Competing interests

The authors declare that they have no competing interests.

## Authors' contributions

The initiative for this work came from AV. Deep well plate and shake flask cultivations for recombinant protein production and subsequent SDS-PAGE analyses were performed by KU and MK. KU, MK and AV drafted the manuscript. TH was responsible for production of recombinant MFE-2 in shake flasks and subsequent protein purification and analysis; TG participated in planning and interpretation of the results. AN participated to research planning, interpretation of results and drafting of the manuscript. MR has participated in planning and setup of the experiments. PN and AV have been advising the medium development, especially optimization of enzymatic glucose delivery and pH maintenance systems demonstrated in this paper. All authors have read and approved the final manuscript.
